# Special Preachers

**Published:** 1890-12-13

**Authors:** 


### Passing Topics.

### SPECIAL PREACHERS.

At the recent meeting of the Council of the Hospital Sunday Fund, the Venerable Archdeacon of London struck the right note when he suggested that special preachers should be obtained for Hospital Sunday in order that the claims and merits of the institutions might be adequately presented to the congregations. This is precisely what is wanted, and what, except in certain notable instances, has never been forthcoming.

We do not hesitate to assert that the Fund is no larger than it is because of the lack of cordial interest displayed by a large majority of the preachers in regard to the object for which they plead. Indeed, some of the clergy do not plead at all. They are content to make a simple announcement at the end of their sermons that the collection of the day will be in aid of the Fund, and having said that, they appear well satisfied that they have done all that is required of them, and that the Fund may be left to take care of itself. In other cases an absolute incapacity to rise to the level of a great subject is distressingly displayed. A more or less appropriate text and a few platitudes dragged in at the tail of the discourse are supposed to be ample, and if anything more is attempted, we get too often an illustration of profound ignorance of hospital work, or a method of handing the subject which is altogether ineffective.

We have long ago ventured to form an opinion that sermons generally would be infinitely more useful and attractive if they were invested with something of a local, or what we may term timely, colour. Nothing arrests the attention of a congregation so much as a reference to a matter to which circumstances lend a personal or present interest. The substance of eternal truth may be woven from mundane materials, and the events of the week or of a neighbourhood may be readily adapted to the furtherance of religion to an extent undreamed of by preachers, who are fonder of theological disquisitions and doctrinal lectures than of simple appeals which strike home to the hearts of a congregation. Homely topics were not beneath the notice of the Divine Teacher, but became as they passed His lips instinct with spiritual life.

Of all the themes which could inspire the pulpit, the great work of healing the sick, which occupied so large a portion of Christ's time on earth, is surely among the noblest, and the most easily comprehended. The dullest performer could scarcely touch the strings attuned to every heart without awakening some responsive notes, and one reason why Hospital Sunday ought to be welcome is that it gives religion a matchless opportunity to show itself in touch with humanity. Let the clergy ponder upon this. Where the Church fails most, it is most out of sympathy with human life. The outcry against sermons would never have arisen did not the preacher too often offer dry bones where the soul asks meat. Doctrine and theology may exercise the intellect of a few, but did they ever teach the many that religion of love whose real power is shown in its ability to penetrate the core of worldly concerns and to make each little duty of life a stepping-stone to higher things?

The privilege of ministering to the temples of the sick is a heritage many of the clergy are indifferent to. Their duty to the sick poor is no more discharged by means of a lifeless sermon than by making use of the hospitals for their parishioners. The hospitals suffer through ignorance of their well-doings. Day and night the great work goes on in splendid silence, leaving no record for the world. The clergy, who send thousands of patients, ought to qualify as ambassadors. A walk through the neighbouring hospital would inspire them with a message and teach them how to plead. Religion, no less than the cause of the sick, will be the gainer when Hospital Sunday is accorded high place in the Church's calendar.

Agricola.

